# Lightning Strikes Twice in the Same Place: Mitochondrial Myopathy and Malignant Hyperthermia—A Case Report

**DOI:** 10.1155/crnm/5802797

**Published:** 2026-09-29

**Authors:** Rafaela Souto e Souza de Melo, Jorge Kiyoshi Mitsunaga Júnior, André Macedo Serafim da Silva, Brandow Willy Souza, Joilson Moura Santos, Pamela Vieira Andrade, Acary Souza Bulle Oliveira, Claudia Ferreira da Rosa Sobreira, Mariz Vainzof, Helga Cristina Almeida da Silva

**Affiliations:** ^1^ Department of Anesthesiology, Federal University of São Paulo, São Paulo, Brazil, unifesp.br; ^2^ Department of Neurology, University of São Paulo, São Paulo, São Paulo, Brazil, usp.br; ^3^ Department of Genetics and Evolutionary Biology, University of São Paulo, São Paulo, Brazil, usp.br; ^4^ Department of Neurology, University of São Paulo, Campus Ribeirao Preto Ribeirao Preto, São Paulo, Brazil, usp.br

## Abstract

Mitochondrial disease predominantly affects tissues reliant on aerobic metabolism, with an incidence of 1 in 4000 individuals and a broad spectrum of clinical presentations. Malignant hyperthermia is a pharmacogenetic disease in which a hypermetabolic reaction is triggered in malignant hyperthermia‐susceptible patients by specific anesthetic agents. We present a clinical case of a fatal atypical reaction to anesthesia, where mitochondrial dysfunction was identified in the mother, and a variant in the *RYR1* gene was found in the asymptomatic father and a paternal first cousin with exercise‐induced rhabdomyolysis. Our report highlights that patients with complex phenotypes may present with concomitant diseases, necessitating comprehensive and often competing management priorities.

## 1. Introduction

In addition to many other metabolic actions, mitochondria are responsible, at the level of their inner membrane, for oxidative phosphorylation, which produces energy in the form of adenosine triphosphate (ATP). That is why mitochondrial diseases predominantly affect systems that rely on aerobic metabolism, such as the nervous system and cardiac/skeletal muscle [1]. Mitochondrial disorders have an incidence of 1:4000 individuals and exhibit variable clinical presentations [1–3]. Heteroplasmy (presence of more than one type of mitochondrial DNA within a single cell) and the threshold effect (impairment of energy production only after the proportion of mutated mitochondrial DNA surpasses a critical limit) explain why patients carrying the same genetic variant may present with different clinical phenotypes and variable responses to anesthesia [1]. As a result of this clinical heterogeneity, their diagnosis is based on family history, biomarkers (e.g., lactate levels), muscle biopsy, and biochemical/molecular investigation [3]. The involvement of multiple organs with no direct relationship (e.g., hearing and heart and bone marrow and pancreas) is also a frequent clinical feature that may raise the suspicion of a mitochondrial disease, although some individuals may remain asymptomatic [3–5].

Abnormal reactions to anesthesia in mitochondrial diseases may result from an imbalance between ATP supply/consumption and may be associated with factors such as metabolic and surgical stress, hypoxemia, and hypoglycemia [6]. Despite these concerns, and unless severe organ damage is present (e.g., cardiomyopathy, renal disease, and severe myopathy), patients with mitochondrial myopathies were not considered at greater risk for anesthesia, and halogenated anesthetics were deemed safe. However, there are concerns regarding propofol infusion syndrome [7], and, recently, sevoflurane [5].

Malignant hyperthermia (MH) is a hypermetabolic reaction triggered in MH‐susceptible (MHS) patients by halogenated agents/succinylcholine due to increased muscular calcium release. MHS patients can be asymptomatic in daily life or present with myopathies, mostly central core disease [8]. While the incidence of the MH crisis is estimated to be 1:10.000–50.000 anesthetic procedures, the frequency of associated mutations can reach 1:870 individuals [7, 8]. MHS diagnosis is established through a positive in vitro contracture test (IVCT) and/or genetic testing [8]. Patients with mitochondrial myopathies are not considered at greater risk of MH than the general population [7].

Our objective was to report a clinical case of fatal atypical reaction to anesthesia leading to the identification of a complex neuromuscular disorder with mitochondrial involvement and a variant in the *RYR1* gene.

## 2. Case Report

A 2‐year‐old boy, weighing 11 kg, ASA Physical Status 1, with adenoid hypertrophy and secretory otitis media, underwent an adenoidectomy and bilateral tympanostomy with ventilation tube insertion in another institution in 2007. In the preanesthetic evaluation, no complementary investigations were performed despite the abnormalities identified on physical examination. The patient presented several congenital anomalies—palpebral ptosis, strabismus, internal cleft palate, pectus carinatum, and cryptorchidism. On the day of the surgery, the patient was monitored with an electrocardiogram, pulse oximeter, and noninvasive blood pressure. Induction was performed with a mixture of sevoflurane/nitrous oxide. After venous puncture, a 5% dextrose solution was initiated, and midazolam and propofol were administered, followed by orotracheal intubation. The patient was maintained on spontaneous ventilation with sevoflurane/nitrous oxide. Eighty minutes after the onset of anesthesia, the patient developed sudden hand/neck stiffness, hyperthermia (43°C, peaking at 45°C), and a cardiac arrhythmia (sinus tachycardia). No hyperventilation was noticed. MH was suspected; sevoflurane was discontinued; and total intravenous anesthesia was performed with midazolam, fentanyl, and propofol. Management included hyperventilation with 100% oxygen, active cooling, intravenous lactated Ringer’s solution, and administration of dantrolene sodium (total dose: 16 mg/kg), along with atropine, epinephrine, 10% calcium gluconate, furosemide, sodium bicarbonate, lidocaine, dipyrone, and dexamethasone. Despite these measures, four cardiac arrests (sinus pauses) developed and, 90 minutes later, the patient died. Venous blood gas analyses obtained during cardiac arrest indicated acidosis (pH 6.8, PaCO2 115 mmHg, PaO2 38 mmHg, BE. −16.8 mmol/L, HCO3 17.7 MEq/L), hyperkalemia (9.8 mEq/L), hyponatremia (117 mEq/L), and hyperlactatemia (102 mg/dL). MH clinical grade scale (CGS) [9] was 66 points (muscle rigidity: 15 points; arterial PaCO_2_ > 60 mmHg: 15 points; arterial pH < 7.25: 10 points; arterial base excess more negative than −8 mEq/L: 10 points; temperature > 38.8°C: 10 points; arrhythmia: 3 points; serum potassium > 6 mEq/L: 3 points), corresponding to Rank 6 (“almost certain”) for the likelihood of a MH episode. The postmortem histopathological examination of the heart revealed no significant morphological abnormalities in the cardiomyocytes and did not identify any specific pathology that could explain the cause of death. DNA from the patient was not available for molecular investigation. Given the absence of a definitive diagnosis and the presence of strabismus and ptosis, which were suggestive of a neuromuscular disorder, a comprehensive neurological evaluation of the family was pursued, and the family was sent for investigation at the MH center in 2011.

The 44‐year‐old mother reported chronic exercise intolerance, myalgia, and cramps. On physical examination, she presented with palpebral ptosis and micrognathia. Laboratory tests revealed elevated creatine kinase (CK) levels (218 IU/L, normal range up to 170 IU/L), normal serum lactate, and slightly reduced forced vital capacity (88%). Electroneuromyography showed a myopathic pattern, and IVCT for MH was negative. Muscle biopsy with histochemistry disclosed a mitochondrial myopathy (Figures 1A–C) showing ragged red fibers, nuclear centralization in 10% of the fibers, a predominance of fiber Type 1 (59%), and COX‐negative fibers (5%). An enzymatic assay of the respiratory chain demonstrated deficiencies in Complexes III and IV. Molecular analysis of mitochondrial and nuclear DNA did not reveal large rearrangements or other pathogenic mutations. The 56‐year‐old father declined further investigations beyond molecular testing. He carried a nonsynonymous single nucleotide variant in the *RYR1* gene (NM_000540/NM_001042723: Exon 51: c.G8197T:p.G2733C), which was absent in all databases. This variant was classified as likely pathogenic according to the ACMG criteria and as a variant of uncertain significance (VUS) by the ClinGen MHS Variant Curation Expert Panel.

**FIGURE 1:**
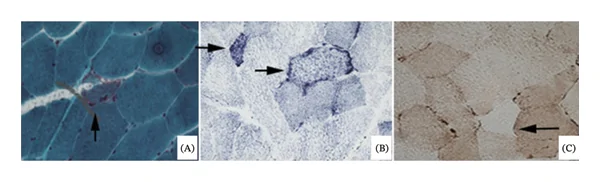
A, B, C: Muscle biopsy of Patient no. 1 (mother). (A) Ragged red fiber (arrow). Frozen transverse section, modified Gomori trichrome staining, 400x. (B) Subsarcolemmal mitochondrial proliferation (arrows). Frozen transverse section, SDH histochemical reaction, 400x. (C) Muscle fiber without cytochrome *c* oxidase (COX) activity (arrow). Frozen transverse section, histochemical reaction COX, 400x.

Further investigation in the family revealed that a 30‐year‐old female paternal first cousin was previously evaluated in another service due to a 10‐year history of exercise intolerance, myalgia, cramps, and recurrent episodes of myoglobinuria (approximately 10 per year). She exhibited mild upper limb muscle weakness, CK levels ranging from 559 to 2376 IU/L, and DNA analysis evidenced the same variant as her uncle.

## 3. Discussion

The diagnostic evaluation of neuromuscular disorders presenting with atypical anesthetic reactions remains a major clinical challenge, particularly when different pathological mechanisms may coexist within the same family [7]. In such scenarios, the interaction between metabolic, structural, and pharmacogenetic factors can lead to unusual phenotypes, complicating both perioperative management and the postevent investigation [1]. In this case, the index patient exhibited multiple dysmorphic features that, though not pathognomonic, are reported both in myopathies and MHS individuals. These findings were not linked to a defined disorder before the anesthetic event, highlighting the difficulty of neuromuscular diagnosis. In addition, his atypical reaction to anesthesia could be related to underlying subclinical myopathy, possibly resulting from mixed inheritance from both parents. During anesthesia, the hypermetabolism state induced by his possible MH crisis may have increased ATP consumption beyond the production capacity, potentially contributing to the fatal outcome despite appropriate dantrolene treatment.

MH crisis typically presents with tachycardia, tachypnea, hypercarbia, muscle rigidity, hyperthermia, acidosis, and rhabdomyolysis [8]. The diagnosis is clinical and represents a challenge, especially in an atypical crisis [7, 8]. Mitochondrial myopathies can lead to atypical reactions during anesthesia, due to mitochondrial abnormalities [1, 7]. Energy production in muscle tissue may be insufficient to supply the demands of metabolic stress [1]. While surgical procedures may cause only mild stress in healthy individuals, they can significantly worsen the clinical status of patients with mitochondrial myopathy [10]. In addition, these patients may have several clinically associated problems (cardiac dysfunction, arrhythmias, ventilatory insufficiency, dysphagia, metabolic decompensation with lactic acidosis, rhabdomyolysis, and sensitivity to anesthetics) [2].

We identified three other reports of patients with concomitant mitochondrial myopathy and MHS who underwent anesthesia [2, 6, 11]. Pepper et al. [2] described a patient with a myopathic phenotype and chronic fatigue, who successfully underwent regional anesthesia. This patient had a possible mitochondrial disease due to reduced Complex I activity, and a possible MH susceptibility due to a VUS in the *RYR1* gene. Etcharry‐Bouyx et al. [11] reported a patient who tolerated multiple general anesthetics procedures uneventfully before experiencing episodes of exertional rhabdomyolysis. He was later diagnosed with MHS after an IVCT and presented a mitochondrial disease based on muscle biopsy findings of COX‐negative fibers and complex IV deficiency. Nelson et al. [6] described two cases. The first patient carried a pathogenic *RYR1* gene variant associated with two variants in mitochondrial disease–related genes. The second patient had a history of two MH‐like crises and a mutation linked to mitochondrial diseases. Both patients successfully underwent general anesthesia without MH‐triggering agents or propofol.

The anesthetic management of patients with coexisting conditions is complex. MHS patients require a properly prepared anesthetic machine, dantrolene availability, and a trigger‐free anesthetic approach [8]. Perioperative optimization for mitochondrial disease patients involves minimizing fasting periods, fluid selection, and strict temperature control to reduce metabolic stress [1, 10]. Perioperative monitoring of temperature, lactate, and blood gases is recommended [1]. There is no universally defined safest anesthetic technique for patients with mitochondrial disease, and all approaches must be used with caution [1, 7]. Only succinylcholine should be avoided due to the risk of rhabdomyolysis [7]. Nondepolarizing neuromuscular blocking agents may be used with caution due to a possibility of prolonged action in neuromuscular disorders [7]. Special attention is required when using propofol due to the risk of propofol infusion syndrome. In patients without mitochondrial diseases, it is recommended to avoid propofol infusion rates exceeding 5 mg/kg/h for more than 48 h; use propofol in combination with other sedative agents (such as opioids); and monitor pH, lactate, and CK levels [12]. In patients with suspected mitochondrial dysfunction, avoiding propofol infusions or limiting its use to short procedures, with adequate glucose supply, should be considered, with alternative agents such as ketamine, remimazolam, or dexmedetomidine used when appropriate [10, 12].

In cases of atypical adverse reactions during anesthesia, it is crucial to investigate the proband and both parents regarding neuromuscular disorders. If the proband dies, investigating both parents alone cannot fully confirm or rule out the MH susceptibility, as the index case may harbor a new *(de novo)* mutation [4, 5]. In our case, the mother tested negative for MH. However, additional muscle histopathological analysis suggested mitochondrial myopathy, later confirmed by biochemical studies. Her normal lactate level is a finding reported in some patients with mitochondrial myopathy [13]. In atypical anesthetic reactions, a negative MH investigation raises the possibility of other diagnoses that may cause complications during anesthesia [7]. The father’s genetic study revealed an *RYR1* variant, with variable clinical presentations. While he was asymptomatic, her niece exhibited exertional rhabdomyolysis, and his child presented dysmorphic features and an atypical reaction to anesthesia. This variant has not been previously described, but molecular analysis strongly suggests that it can be pathogenic.

Unfortunately, no blood or tissue samples were collected at the time of death. Such samples are important in atypical reactions during anesthesia and would have been essential for confirming, with molecular and biochemical tests, the diagnosis of mitochondrial disease and MH in the child.

Our report highlights that patients with complex phenotypes may present with overlapping conditions that require a multidisciplinary approach with competing management priorities. Neurologists, anesthesiologists, geneticists, and pathologists must work collaboratively, from preoperative risk stratification to postevent investigation, to ensure accurate diagnosis, guide management strategies, and inform genetic counseling. Early recognition of possible underlying neuromuscular disorders is crucial, as it may allow tailored perioperative planning and potentially prevent fatal outcomes.

## Funding

This study was supported by theCoordination for the Improvement of Higher Education Personnel, 001, and the São Paulo Research Foundation, 2021/06180‐7.

## Disclosure

This work was presented at the 43rd EMGH Meeting 2025.

## Consent

Written informed consent was obtained from the patient’s family for publication of this case report and any accompanying images.

## Conflicts of Interest

The authors declare no conflicts of interest.

## Data Availability

The data that support the findings of this study are available on request from the corresponding author. The data are not publicly available due to privacy or ethical restrictions.
